# Markedly Elevated Liver Enzymes in Choledocholithiasis in the absence of Hepatocellular Disease: Case Series and Literature Review

**DOI:** 10.1177/2324709616651092

**Published:** 2016-05-18

**Authors:** Eula Plana Tetangco, Natasha Shah, Hafiz Muhammad Sharjeel Arshad, Hareth Raddawi

**Affiliations:** 1 University of Illinois at Chicago, IL, USA; 2 Advocate Christ Medical Center, Oak Lawn, IL, USA

**Keywords:** hepatitis, transaminitis, choledocholithiasis, gallstones

## Abstract

Liver enzyme levels are commonly obtained in the evaluation of many conditions. Elevated alanine transaminase and aspartate transaminase have traditionally been considered a “hepatocellular” pattern concerning for ischemic, viral, or toxic hepatitis. Elevations in these levels pose a diagnostic dilemma in patients without a clinical picture consistent with liver disease. On the other hand, elevated alkaline phosphatase historically represents a “cholestatic” pattern concerning for gallbladder and biliary tract disease. Often, patients present with a “mixed” picture of elevation in all 3 liver enzymes, further confounding the clinical scenario. We present 4 cases of women with severe upper abdominal pain and markedly elevated transaminases. Three of the patients had accompanying jaundice. A higher rise in enzyme levels was seen in those who had greater bile duct dilation. All patients saw a rapid decrease in transaminases after biliary decompression, along with a fall in alkaline phosphatase and total bilirubin levels. No evidence of liver disease was found, nor were there any signs of hepatocellular disease on imaging. The patients were ultimately found to have choledocholithiasis on endoscopic retrograde cholangiopancreatography with no hepatocellular disease. Furthermore, our cases show that severe abdominal pain in the setting of elevated liver enzymes is likely associated with biliary disease rather than a primary hepatic process. Recognition of this rare pattern of markedly elevated transaminases in isolated biliary disease can aid in avoiding unnecessary evaluation of primary hepatic disease and invasive surgical interventions such as liver biopsy.

## Introduction

Liver enzyme levels are commonly obtained in the evaluation of many conditions. Alanine transaminase (ALT, formerly serum glutamic-pyruvic transaminase or SGPT) is produced in hepatocytes and is a sensitive marker for liver injury. Aspartate transaminase (AST, formerly serum glutamic-oxaloacetic transaminase or SGOT) comes in 2 isoenzymes. The mitochondrial isoenzyme, like ALT, is produced in hepatocytes. The cytosolic isoenzyme is produced in skeletal and heart muscle, as well as the kidney. Alkaline phosphatase (ALP) is produced mainly in the biliary epithelium, with some isoenzymes being produced in kidney, intestine, leukocytes, placenta, and bone. Release is triggered by bile salt accumulation or cholestasis. A physiologic rise is also seen in pregnancy and in growing children.^1^ Elevated ALT and AST levels have traditionally been considered a “hepatocellular” pattern, concerning for ischemic, viral, or toxic hepatitis. In contrast, elevated ALP gives the “cholestatic” pattern of biliary and biliary tract disease. Often, patients present with a “mixed” picture of elevation in all 3 liver enzymes.^2,3^ Laboratory findings are an adjunct to thorough history and physical examination. In many cases, however, the presenting symptoms are nonspecific, further confounding the mixed picture of liver enzymes. We present 4 cases of women with severe upper abdominal pain and markedly elevated transaminases, all of whom were ultimately found to have choledocholithiasis on endoscopic retrograde cholangiopancreatography (ERCP) with no hepatocellular disease (see Table 1).

**Table 1.** Initial and Follow-up Liver Tests in 4 Patients With Isolated Biliary Disease.

	Age (Years)	AST (units/L)		ALT (units/L)		ALP (units/L)		Bilirubin (mg/dL)	
		Initial	Follow-up	Initial	Follow-up	Initial	Follow-up	Initial	Follow-up
Patient 1	89	1,134	32	1,211	73	215	84	4.2	1.0
Patient 2	89	1,110	26	590	265	23	160	1.6	0.4
Patient 3	76	1,023	33	974	27	117	53	4.4	1.3
Patient 4	28	634	199	1,036	568	93	86	6.9	1.3

Abbreviations: AST, aspartate transaminase; ALT, alanine transaminase; ALP, alkaline phosphatase.

## Case 1

An 89-year-old female with no chronic medical problems was evaluated for severe upper abdominal pain following a meal of beef stew. The pain lasted several hours and was associated with nausea and vomiting. On admission she was afebrile, with a blood pressure of 113/68 mm Hg, heart rate of 89 beats per minute, and respiratory rate of 18 breaths per minute. Physical examination revealed icteric sclerae, mild right upper quadrant tenderness, and no abdominal masses or organomegaly. Laboratory evaluation was significant for AST of 1134 units/L, ALT of 1211 units/L, alkaline phosphatase of 215 units/L, total bilirubin of 4.2 mg/dL, and normal lipase and amylase levels. An abdominal ultrasound demonstrated a partially distended gallbladder, cholelithiasis, intrahepatic duct dilation, and common bile duct (CBD) with diameter of 8 mm with no apparent choledocholithiasis. ERCP was performed revealing a markedly dilated bile duct containing a large 15 × 20 mm stone, and a narrow bile duct outlet concerning for ampullary stenosis. Biliary sphincterotomy and dilation were performed, recovering a large amount of biliary sludge, but with inability to extract the stone. Due to episodes of atrial fibrillation, the procedure was terminated in favor of placing a temporary 5-French 4-cm double pigtail biliary stent for large burden stone disease. The patient’s pain and icterisia resolved and hepatic chemistries trended down.

## Case 2

An 89-year-old female was seen for sudden-onset upper abdominal pain. She had a history of cholecystitis that was treated conservatively with a cholecystostomy tube, which was subsequently removed a few months later. Her other medical problems included peptic ulcer disease, congestive heart failure, diabetes mellitus, hypertension, paroxysmal atrial fibrillation, and dementia. She was afebrile, with a blood pressure of 95/56 mm Hg, heart rate of 71 beats per minute, and respiratory rate of 17 breaths per minute. On exam she had some mild gastric tenderness. Laboratory studies revealed AST of 1110 units/L, ALT of 590 units/L, alkaline phosphatase of 153 units/L, and total bilirubin of 1.6 mg/dL. Abdominal ultrasound showed cholelithiasis and sludge is present with an unremarkable CBD and liver. On ERCP she had a 9-mm bile duct without obvious filling defects. Sphincterotomy and balloon sweeps were performed, with removal of scant amount of yellow debris. No stones were seen. Excellent bile flow was noted. Symptoms improved and hepatic chemistries normalized.

## Case 3

A 76-year-old female was admitted for severe upper abdominal pain that woke her from sleep. She had a history of breast cancer, hyperlipidemia, diverticulitis, and constipation. She was afebrile on admission with a blood pressure of 88/56 mm Hg that responded to fluid resuscitation, heart rate of 60 beats per minute with an irregular rhythm, and respiratory rate of 16 breaths per minute. On exam she was jaundiced, with mild epigastric and right upper quadrant tenderness, no abdominal masses or organomegaly. Laboratory evaluation was significant for AST of 1023 units/L, ALT of 974 units/L, alkaline phosphatase of 117 units/L, total bilirubin of 4.4 mg/dL, and lipase of 5470 units/L that normalized after a few hours. She had no leukocytosis, and serologic hepatitis studies were negative. Abdominal ultrasound demonstrated cholelithiasis with gall bladder sludge and wall thickening and borderline prominent intrahepatic bile ducts. Noncontrast abdominal computed tomography was consistent with cholelithiasis. ERCP revealed a slightly prominent bile duct with amorphous filling defects and a narrow outlet consistent with ampullary stenosis. Stones were seen in the gallbladder, with no evidence of cystic duct obstruction. Successful 12-mm endoscopic sphincterotomy and balloon sweep was performed. Biliary sludge and tiny stone particles were extracted, with suggestion of bile duct microlithiasis. Free flow of bile and contrast from the bile duct into the duodenum was clearly established. Abdominal pain resolved and transaminases trended down, normalizing on 1-month follow-up.

## Case 4

A 28-year old female with no significant past medical history was evaluated for sudden-onset upper abdominal pain associated with nausea. She was afebrile, with a blood pressure of 128/83 mm Hg, heart rate of 57 beats per minute, and respiratory rate of 18 breaths per minute. On exam sclerae were slightly icteric, and she had mild epigastric and right upper quadrant tenderness. Laboratory evaluation revealed AST of 634 units/L, ALT of 1036 units/L, ALP of 94 units/L, and total bilirubin of 6.9 units/L. Serologic testing for hepatitis A, B, and C were negative. Abdominal imaging was consistent with cholelithiasis. ERCP revealed 3 stones in the CBD that were each 12 mm in size. Sphincterotomy resulted in extraction of stones with excellent drainage. Symptoms resolved and hepatic chemistries normalized.

## Discussion

High levels of serum aminotransferases are used as indicators of liver parenchymal disease. AST and ALT are specific to the liver, with levels greater than 400 units/L being indicative of hepatocellular disease. Levels above 1000 units/L raise suspicion for processes that cause necrosis of hepatocellular tissue such as ischemia, viral hepatitis, or drug toxicity.^2^ Markedly elevated AST levels have also been described in acute pancreatitis and pancreatic carcinoma.^3^ Rarely, isolated calculous disease of the biliary tract with no hepatic disease or involvement can present with markedly elevated AST and ALT levels. It is an underrecognized phenomenon that enzymes can reach similar levels in patients with symptomatic choledocholithiasis.

Transaminase levels of about 500 units/L in both cholelithiasis and choledocholithiasis have been reported as early as 1985, with Fortson et al^3^ examining 9 patients with extrahepatic biliary tract disease having liver enzymes above 600 units/L. Anciaux et al^4^ found that the incidence and enzyme levels were higher in those with CBD stones, and there was decrease in enzyme levels to near-normal levels 2 to 4 weeks after cholecystectomy. One other study by Nathwani et al^2^ noted hepatic chemistries elevated above 1000 units/L.

In such cases, the etiology behind remarkably elevated liver enzymes greater than 1000 units/L remains unclear. Experiments with bile duct ligation in dogs have produced AST levels in the several thousands, with higher levels seen after cholecystectomy.^2^ Proposed mechanisms include elevated bile duct pressures due to the stone causing reflux of liver enzymes into hepatic sinusoids, increased production of transaminases, increased permeability of hepatocytes to release enzymes into the bloodstream, and bile acid radicals that have a direct toxic effect leading to apoptosis and eventual liver necrosis.^5,6^

All our patients presented with acute upper abdominal pain, and 3 patients had jaundice. Pain has been cited as the most frequent symptom of choledocholithiasis, usually with accompanying symptoms.^4^ Higher enzyme levels were seen with in greater degrees of bile duct dilation. In all our cases, transaminases rapidly decreased after intervention, along with a fall in alkaline phosphatase and total bilirubin levels. This rapid fall of transaminases distinguishes these cases from primary hepatocellular disease.^6^ No evidence of liver disease characterized by persistent elevation in enzymes was found, nor was there any suspicion for hepatocellular disease on imaging. It was not indicated to continue extensive workup in search of a coexisting disease that could cause the elevated levels. Furthermore, our cases show that severe abdominal pain in the setting of elevated liver enzymes is likely associated with biliary disease rather than a primary hepatic process. Recognition of this rare pattern in isolated biliary disease can prevent unnecessary evaluation of primary hepatic disease and invasive diagnostic modalities such as liver biopsy.

In summary, isolated choledocholithiasis can result in transient but profoundly elevated transaminase levels that fall rapidly after the primary biliary disease is addressed and ductal clearance is achieved through methods such as ERCP. Recognition and familiarity of such a rare pattern can assist physicians in diagnosis and prevents unnecessary evaluation of primary hepatic disease or invasive modalities such as liver biopsy.
